# Supplementary material for: Workplace-Based Interventions for Mental Health in Africa: A Scoping Review
Source: Int J Environ Res Public Health. 2023 May 18;20(10):5863. doi: 10.3390/ijerph20105863 (PMC10218638; doi:10.3390/ijerph20105863)
Supplement: Supplementary file 1 [file ijerph-20-05863-s001.zip › ijerph-2325124-supplementary.pdf]

Pubmed:

No limiters applied.

("Mental Disorders"[Mesh] OR "Mental Health"[Mesh] OR "Mentally Ill Persons"[Mesh] OR "mental health" OR "mental disorder*" OR burnout OR stress OR psychosocial OR wellbeing OR well-being OR wellness OR "substance abuse" OR "alcohol abuse" OR "drug abuse" OR "post traumatic stress disorder" OR "post-traumatic stress disorder" OR PTSD OR depression OR anxiety OR schizophrenia OR suicide) AND (Rehabilitation[Mesh] OR "occupational therap*" OR rehabilitation OR prevention OR promotion OR habilitation OR "supported employment" OR return-to-work OR "return to work" OR counsel* OR mindfulness OR nature-based OR therapy OR treatment OR intervention OR effect* OR evidence-based) AND (Workplace[Mesh] OR Employment[Mesh] OR workplace OR worksite OR work-site OR "work site" OR "job site" OR job-site OR employment) AND (Africa[Mesh] OR Africa* OR Botswana OR Ghana OR Kenya OR Madagascar OR Malawi OR Mauritius OR Morocco OR Namibia OR Nigeria OR Rwanda OR Seychelles OR "South Africa*" OR Tanzania OR Tunisia OR Uganda OR Zambia OR Zimbabwe)

Web of Science:

No limiters applied

TOPIC: (("Mental Disorders" OR "Mental Health" OR "Mentally Ill Persons" OR "mental health" OR "mental disorder*" OR burnout OR stress OR psychosocial OR wellbeing OR well-being OR wellness OR "substance abuse" OR "alcohol abuse" OR "drug abuse" OR "post traumatic stress disorder" OR "post-traumatic stress disorder" OR PTSD OR depression OR anxiety OR schizophrenia OR suicide) AND (Rehabilitation OR " occupational therap*" OR rehabilitation OR prevention OR promotion OR habilitation OR "supported employment" OR return-to-work OR "return to work" OR counsel* OR mindfulness OR nature-based OR therapy OR treatment OR intervention OR effect* OR evidence-based) AND (Workplace OR Employment OR workplace OR worksite OR work-site OR "work site" OR "job site" OR job-site OR employment) AND (Africa OR Africa* OR Botswana OR Ghana OR Kenya OR Madagascar OR Malawi OR Mauritius OR Morocco OR Namibia OR Nigeria OR Rwanda OR Seychelles OR "South Africa*" OR Tanzania OR Tunisia OR Uganda OR Zambia OR Zimbabwe))

Timespan: All years. **Indexes:** SCI-EXPANDED, SSCI, A&HCI, CPCI-S, BKCI-S, BKCI-SSH, ESCI, IC.

Ebscohost

- Academic Search Premier (1,295)
- ☐ MEDLINE (1,281)
- ☐ Africa-Wide Information (943)
- ☐ CINAHL (855)
- ☐ Health Source: Nursing/Academic Edition (310)

((MH "Mental Disorders+") OR (MH "Mental Health+") OR (MH "Mentally Ill Persons+") OR "mental health" OR "mental disorder*" OR burnout OR stress OR psychosocial OR wellbeing OR well-being OR wellness OR "substance abuse" OR "alcohol abuse" OR "drug

abuse" OR "post traumatic stress disorder" OR "post-traumatic stress disorder" OR PTSD OR depression OR anxiety OR schizophrenia OR suicide) AND ((MH "Rehabilitation+") OR "occupational therap*" OR rehabilitation OR prevention OR promotion OR habilitation OR "supported employment" OR return-to-work OR "return to work" OR counsel* OR mindfulness OR nature-based OR therapy OR treatment OR intervention OR effect* OR evidence-based) AND ((MH "Workplace+") OR (MH "Employment+") OR workplace OR worksite OR work-site OR "work site" OR "job site" OR job-site OR employment) AND ((MH "Africa+") OR Africa* OR Botswana OR Ghana OR Kenya OR Madagascar OR Malawi OR Mauritius OR Morocco OR Namibia OR Nigeria OR Rwanda OR Seychelles OR "South Africa*" OR Tanzania OR Tunisia OR Uganda OR Zambia OR Zimbabwe)

Cochrane

([mh "Mental Disorders"] OR [mh "Mental Health"] OR [mh "Mentally Ill Persons"] OR "mental health" OR ("mental" NEAR/2 disorder*) OR burnout OR stress OR psychosocial OR wellbeing OR well-being OR wellness OR "substance abuse" OR "alcohol abuse" OR "drug abuse" OR "post traumatic stress disorder" OR "post-traumatic stress disorder" OR PTSD OR depression OR anxiety OR schizophrenia OR suicide) AND ([mh Rehabilitation] OR ("occupational" NEAR/2 therap*) OR rehabilitation OR prevention OR promotion OR habilitation OR "supported employment" OR return-to-work OR "return to work" OR counsel* OR mindfulness OR nature-based OR therapy OR treatment OR intervention OR effect* OR evidence-based) AND ([mh Workplace] OR [mh Employment] OR workplace OR worksite OR work-site OR "work site" OR "job site" OR job-site OR employment) AND ([mh Africa] OR Africa* OR Botswana OR Ghana OR Kenya OR Madagascar OR Malawi OR Mauritius OR Morocco OR Namibia OR Nigeria OR Rwanda OR Seychelles OR ("South" NEAR/2 Africa*) OR Tanzania OR Tunisia OR Uganda OR Zambia OR Zimbabwe)):ti,ab,kw" (Word variations have been searched)

Scopus

(INDEXTERMS("Mental Disorders") OR INDEXTERMS("Mental Health") OR INDEXTERMS("Mentally Ill Persons") OR "mental health" OR "mental disorder*" OR "burnout" OR "stress" OR "psychosocial" OR "wellbeing" OR "well-being" OR "wellness" OR "substance abuse" OR "alcohol abuse" OR "drug abuse" OR "post traumatic stress disorder" OR "post-traumatic stress disorder" OR "PTSD" OR "depression" OR "anxiety" OR "schizophrenia" OR "suicide") AND (INDEXTERMS("Rehabilitation") OR "occupational therap*" OR "rehabilitation" OR "prevention" OR "promotion" OR "habilitation" OR "supported employment" OR "return-to-work" OR "return to work" OR "counsel*" OR "mindfulness" OR "nature-based" OR "therapy" OR "treatment" OR "intervention" OR "effect*" OR "evidence-based") AND (INDEXTERMS("Workplace") OR INDEXTERMS("Employment") OR "workplace" OR "worksite" OR "work-site" OR "work site" OR "job site" OR "job-site" OR "employment") AND (INDEXTERMS("Africa") OR "Africa*" OR "Botswana" OR "Ghana" OR "Kenya" OR "Madagascar" OR "Malawi" OR "Mauritius" OR "Morocco" OR "Namibia" OR "Nigeria" OR "Rwanda" OR "Seychelles" OR "South Africa*" OR "Tanzania" OR "Tunisia" OR "Uganda" OR "Zambia" OR "Zimbabwe")

Otseeker

Work and mental health, advanced search

Sabinet Reference

Sabinet reference, Theses and dissertations, African digital repository
1 result
Sabinet reference, Theses and dissertations, Current and completed research

Mental health workplace
And
Occupational therapy

“mental health” and work – 131 results

Pro Quest

TOPIC: (("Mental Disorders" OR "Mental Health" OR "Mentally Ill Persons" OR "mental health" OR "mental disorder*" OR burnout OR stress OR psychosocial OR wellbeing OR well-being OR wellness OR "substance abuse" OR "alcohol abuse" OR "drug abuse" OR "post traumatic stress disorder" OR "post-traumatic stress disorder" OR PTSD OR depression OR anxiety OR schizophrenia OR suicide) AND (Rehabilitation OR " occupational therap*" OR rehabilitation OR prevention OR promotion OR habilitation OR "supported employment" OR return-to-work OR "return to work" OR counsel* OR mindfulness OR nature-based OR therapy OR treatment OR intervention OR effect* OR evidence-based) AND (Workplace OR Employment OR workplace OR worksite OR work-site OR "work site" OR "job site" OR job-site OR employment) AND (Africa OR Africa* OR Botswana OR Ghana OR Kenya OR Madagascar OR Malawi OR Mauritius OR Morocco OR Namibia OR Nigeria OR Rwanda OR Seychelles OR "South Africa*" OR Tanzania OR Tunisia OR Uganda OR Zambia OR Zimbabwe))

Filters: (mental health) AND (therapy OR health sciences OR health care OR rehabilitation)
